# Printed Circuit Board (PCB) Technology for Electrochemical Sensors and Sensing Platforms

**DOI:** 10.3390/bios10110159

**Published:** 2020-10-30

**Authors:** Hamed Shamkhalichenar, Collin J. Bueche, Jin-Woo Choi

**Affiliations:** 1 Aquatic Germplasm and Genetic Resources Center, School of Renewable Natural Resources, Louisiana State University Agricultural Center, Baton Rouge, LA 70820, USA; hshamk1@lsu.edu; 2 School of Electrical Engineering and Computer Science, Louisiana State University, Baton Rouge, LA 70803, USA; cbuech8@lsu.edu; 3 Center for Advanced Microstructures and Devices, Louisiana State University, Baton Rouge, LA 70803, USA

**Keywords:** printed circuit board, sensor electrode, electrochemical sensor

## Abstract

The development of various biosensors has revolutionized the healthcare industry by providing rapid and reliable detection capability. Printed circuit board (PCB) technology has a well-established industry widely available around the world. In addition to electronics, this technology has been utilized to fabricate electrical parts, including electrodes for different biological and chemical sensors. High reproducibility achieved through long-lasting standard processes and low-cost resulting from an abundance of competitive manufacturing services makes this fabrication method a prime candidate for patterning electrodes and electrical parts of biosensors. The adoption of this approach in the fabrication of sensing platforms facilitates the integration of electronics and microfluidics with biosensors. In this review paper, the underlying principles and advances of printed board circuit technology are discussed. In addition, an overview of recent advancements in the development of PCB-based biosensors is provided. Finally, the challenges and outlook of PCB-based sensors are elaborated.

## 1. Introduction

In general, the primary factors in choosing a desirable detection method and tools for specific applications are cost, sensitivity, reliability, and rapidity. Cost is one of the main driving forces behind modern innovation but not the only important parameter. Obtaining reliable and accurate measurements in a short amount of time cannot always be sacrificed to reduce expenses. In clinical diagnostic applications, the reliability and rapidity of data play important roles. For example, reliable real-time measurement of blood glucose is essential in controlling the progress of diabetes [1].

To develop cost-effective and accurate sensors, the adoption of suitable detection methods, fabrication techniques, and materials required for the development of the sensors should be considered. Electrochemical analyses can offer an economical approach to quantify chemicals and detect changes in the physical characteristic of materials with high selectivity and sensitivity [2]. In terms of equipment, such techniques generally require electrochemical sensors composed of two or three electrodes called working (sensing), reference, and counter (auxiliary) electrodes in addition to electronic instrumentation for collecting data. Although the conventionally required electronic instrumentation can be sizeable and expensive, these devices can be miniaturized using recent advances in electronics. The implementation of such miniaturized instruments can facilitate the utilization of electrochemical sensors in point-of-care and field-deployable applications.

Different fabrication methods can be considered to construct electrochemical sensors. For example, microfabrication techniques used in the semiconductor industry are well established due to their flexibility in the adoption of a vast range of materials and techniques offering outstanding control over the sensors parameters. However, multiple techniques (e.g., sputtering, chemical vapor deposition, photolithography) and specialized facilities may be required to fabricate these sensors [3]. As an alternative approach, printed circuit board technology (PCB) has the potential for the construction of sensors. This technology is a well-established economical manufacturing method widely used to fabricate electronic circuitry. Nowadays, PCB fabrication is broadly available at relatively low cost due to considerable growth in the electronics industry during the past decades [4]. PCB technologies make it possible to pattern conductive electrodes with high precision that can be used as a substrate for sensors. Although PCB technology employs techniques similar to microfabrication processes, it provides widely available affordable manufacturing possibilities.

This review aims to provide an overview of the utilization of PCB technology in the development of electrochemical sensors and miniaturized sensing platforms. To begin with, the background of the printed circuit board technology is discussed, along with recent advances in this field. Next, an overview of the recent advances in the development of PCB-based electrochemical sensors and sensing platforms was provided.

## 2. Printed Circuit Board Technology

### 2.1. History

In 1903, Albert Hanson created the first printed circuit board by laminating flat foil conductors to an insulating board. In 1904, Thomas Edison formed conductors onto linen paper by utilizing patterned polymer adhesives. While the aforementioned designs would be nearly unrecognizable today, Hanson and Edison laid the groundwork for what would become an essential component to the modern electronics industry. From the early 1900s until the 1940s, few advancements were made, and the boards were limited to the usage of only a single side. However, the United States Army began to use PCBs to make proximity fuses in 1943, later releasing the technology to the public after the war [5]. In the 1950s, through-hole technology was the most popular method of mounting electronic components onto a board. Through-hole technology involves mounting the leads of the component in holes drilled on the board and soldering the leads in place from the underside of the board. Because of the need to drill holes into the board, the available space and routing area was always limited when manufacturing using through-hole technology.

Surface-mount technology (SMT) became a mainstream manufacturing technology for electronics on printed circuit boards in the 1980s, leading to a significant reduction in size, cost, and complexity. SMT allows for more components to be placed in the same space compared to through-hole technology due to the fact that no holes have to be drilled. Furthermore, components can be placed on both sides of the board. Most importantly, SMT boards can be fabricated in multiple layers, which makes them a great candidate for the implementation of high-speed electronics by providing precisive control over the impedance of the traces and electromagnetic interference. Regarding current PCB manufacturing technology, SMT is heavily favored over through-hole. However, through-hole is still used for simpler boards and is easier to solder by hand due to the larger size as opposed to SMT boards. The vast majority of boards manufactured today employ surface-mount technology, and anyone can utilize CAD software to create a design, send it to a manufacturer, and have their own PCB constructed.

### 2.2. Materials

The most common printed circuit board substrate is known as FR4 (flame retardant 4). FR4 is a class of materials that meet National Electrical Manufacturers Association Industrial Thermosetting Products (NEMA LI 1-1998) requirements. The basis of FR4 is composed of woven fiberglass cloth combined with an epoxy resin binder that is flame-resistant. FR4 has near-zero water absorption, as well as an excellent strength to weight ratio, and is an excellent insulator regardless of moisture levels in the ambient. Typically, the flame-resistant material in FR4 is bromine. Along with its other aforementioned properties, the reason FR4 is the most popular substrate because it is easy to manufacture and usually the cheapest material available. However, other materials are often used depending on the environment the board will be placed in, the budget, and the required circuit properties.

Polyimide laminates offer improvements in every category over FR4, most importantly, higher temperature performance, electrical performance, survivability, and resistance to expansion. The cost to manufacture polyimides, however, is higher than FR4. Teflon laminates offer improved electrical properties over both FR4 and polyimide-based substrates; however, they cost significantly more to produce than both and require specialized equipment and a highly-skilled workforce. Teflon laminates can be coated onto glass fabric or manufactured as an unsupported mesh, giving it an adaptability factor that neither the FR4 nor the polyimide possesses.

**Figure 1:**
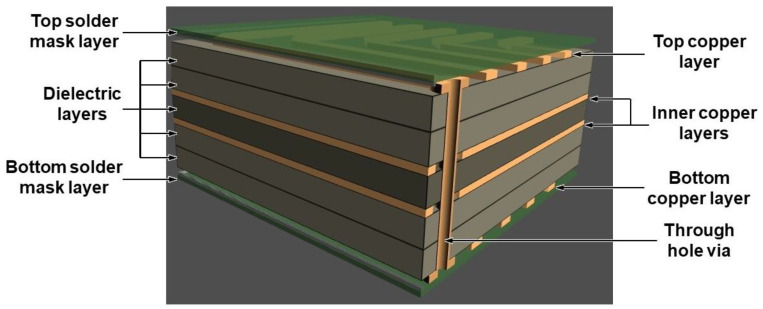
A 3-D schematic design of a multi-layer printed circuit board (PCB) board composed of four copper layers (two internal layers), five dielectric layers, and the top and bottom mask layer.

The multi-layer manufacturing process begins with the creation of a computer-aided design or CAD, which is then sent to the chosen manufacturer. The manufacturer checks to make sure the CAD is compatible with their equipment. A photographic image of the CAD is printed on film, and the image is transferred to the board surface, using photosensitive dry-film and ultraviolet light in a cleanroom. The photographic film is removed, and excess copper is etched from the board. The inner layers receive an oxide layer application and are then stacked with prepreg providing insulation between layers, and copper foil is added to the top and bottom of the newly created stack. An oxide layer application strengthens the laminate bond by increasing the roughness of clad copper. The oxide layer is a chemical composition consisting of compounds such as sodium chlorite (NaClO_2_), water, and sodium hydroxide. The internal layers are laminated by subjecting them to extreme pressure and high temperature. Slowly, pressure is released, and the PCB cures while still at a high temperature. Next, holes are drilled to secure the stack, and excess copper is filed off. A chemical is used to fuse all the layers of the board together, and then the board is cleaned. After cleaning, a series of chemicals bathe the board, resulting in a layer of copper weighing 1 oz/ft^2^ (305.152 g/m^2^), which results in a thickness of 1.4 mil (35 µm), filling in the drill holes and settling on the top layer. Using imperial units such as oz, mil, oz/ft^2^ over metric units is a convention in PCB industry. In addition, oz is often used over oz/ft^2^ to refer copper weight spread evenly over 1 ft^2^ (305.152 g/m^2^) PCB area to determine the copper layer thickness. Once again, the board needs to receive a photoresist application, but only on the outer layers. After the photoresist application, the outer layers are plated the exact same way the inner layers were, but a plating of tin is applied to protect the outer-layer copper from etching. Etching takes place on the outer layers, and excess copper is removed via a copper etchant, with the tin safeguarding the remaining copper. The panels are cleaned and prepared for a solder mask [6]. After cleaning, ink epoxy and solder mask film are applied, and the boards are exposed to ultraviolet light to designate a certain area of the solder mask for removal. The board is baked, allowing the solder mask to cure (Figure 1). The board is plated with gold, silver, or hot air solder level (HASL), enabling the components to be soldered to the pads and to protect the copper. The process by which the board receives plating is known as electroless nickel immersion gold (ENIG). A nickel layer is applied to the copper as a diffusion barrier. Following the nickel layer is a thin gold layer which serves to prevent nickel oxidation and maintains a solid surface of which to solder [7]. After gold or silver-plating, the board is silk-screened, receiving all of the vital information, such as warning labels and company ID numbers. Finally, the board is tested and cut to fit design specifications.

Finishes on the board surface protect exposed copper and provide solderable surfaces. Historically, HASL has been the most prevalent finish in the industry. HASL costs little and is widely available. The circuit boards are immersed in a molten mixture of tin/lead, and excess solder is removed by blowing hot air across the surface of the board. However, the use of ENIG has been rapidly increasing. Nickel forms the layer, which provides a barrier for the copper, as well as being the solderable surface. Gold is then used to protect the nickel and provides the low contact resistance necessary for the thin gold deposits. Electroless nickel electroless palladium immersion gold (ENEPIG), which has been developed relatively recently, has seen increasing usage, despite its high cost because of reliance on palladium [8]. ENEPIG is significantly more resistant to corrosion compared with ENIG and HASL, allowing the PCB to last for a longer period. Across all measurable categories, ENEPIG is superior to HASL and ENIG, but the cost is also noticeably higher. Furthermore, of note is the hard electrolytic gold finish, which consists of a layer of gold plated over a coat of nickel. Hard gold is very durable and used on boards that experience high wear. Hard electrolytic gold is similar to ENIG, but the hard gold layer is generally two to three times as thick (0.005–0.010 mil or 0.127–0.254 µm). In regard to high-wear areas of the board that use the hard gold as a protective layer, the gold can be as thick as 1 mil (25.4 µm), meaning hard electrolytic gold plating is an expensive option.

### 2.3. State-of-the-Art Technology

As PCB technology has progressed over the years, a few key features have gradually improved as well. The most important of these are the number of conducting layers, minimum trace spacing, and minimum trace width. The maximum number of layers most manufacturers will produce is 40. Trace width and spacing are directly proportional to the weight of copper that plates the board. With only 0.5 oz (14.17 g) of copper on the inner layers, trace width can be minimized to 4 mil (101.6 µm). A 1 oz (28.35 g) copper deposit on the outer layers will yield the same 4 mil (101.6 µm) trace width. Distance between traces is identical to width for the majority of manufacturers.

Many applications require cyclic movement or stretching while still maintaining an electrical connection. To fit this need, flexible circuit boards have become an ever-evolving solution. Flexible PCBs or fPCBs have a variety of real-world applications, ranging from laptops to smartphones, engine management units to hard drives. Flexible PCBs are placed in laptops to ensure the connection between the computer and monitor remains intact, as a laptop may be folded thousands of times in its lifetime. Hard drives need to withstand high temperatures and transfer data quickly. Flexible PCBs can also be used as sensors, as on an automobile or even for general purpose. The automobile has many moving parts, and cars today have sensors on every conceivable component. A typical circuit board would not be able to withstand the stretching and bending or the constant change in ambient conditions.

Flexible circuit boards first came about in the early 2000s, with polydimethylsiloxane or PDMS as the most common substrate [5]. However, the most popular substrate materials have since become polyimide film, polyester film, and polyethylene naphthalate (PEN). Polyimide film is the most popular because of its great thermal resistance capabilities and excellent mechanical and electrical properties. High humidity absorption and proneness to tearing harms the polyimide film, but some variants have improved upon these areas. Offering competition is PEN, which fills an intermediate slot in the market. With most qualities’ inferior to that of polyimides, PEN also comes in cheaper. The performance of PEN is still more than adequate for most applications, and it is becoming more and more popular each year. The smallest boards can be as thin as 4 mil (101.6 µm). Trace width on a flexible PCB can be as low as 3 mil (76.2 µm) and spacing as low as 3 mil (76.2 µm). Flex circuits can have multiple layers, up to 10 layers from most manufacturers. The lowest weight of copper is 0.5 oz (14.17 g) but ranges up to 2 oz (56.7 g).

## 3. PCB-Based Electrodes for Electrochemical Analyses

**Figure 2:**
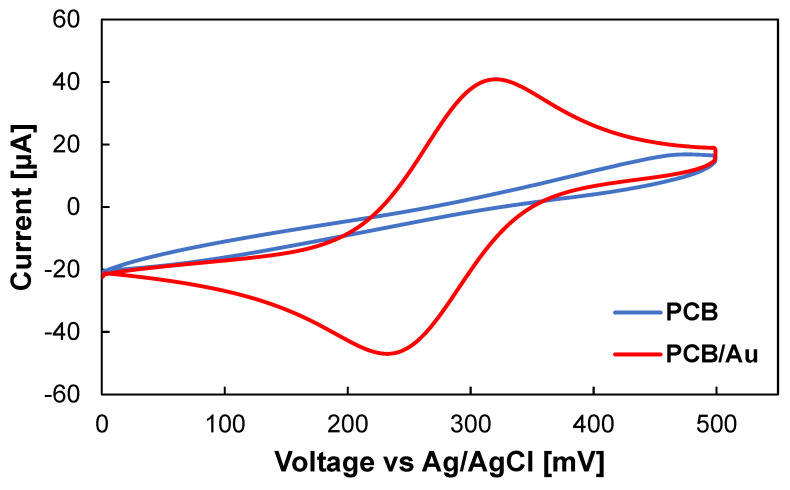
Cyclic voltammetry from −100 to 500 mV vs Ag/AgCl, inside 0.1 M KCl containing 5 mM K_3_Fe(CN)_6_ using PCB (blue line), and Au-plated PCB (red line) electrodes. The scan rate was 50 mV/s.

Copper is the most commonly used material in the fabrication of traces and electric contacts on PCB boards. However, the easy and unavoidable oxidation problem of copper limits its application in developing electrochemical sensors [9]. To overcome this challenge, a thin layer of inert metals, such as gold (Au) or platinum (Pt), can be deposited on the surface of PCB pads. The cyclic voltammetry analysis was performed using bare Cu PCB electrode shows a non-characteristic voltammogram since Cu can easily oxidize. On the other hand, the adoption of the Au-plated PCB electrode results in a stable voltammogram with a wide potential window acceptable for electrochemical biosensing applications (Figure 2). Different techniques can be employed to deposit gold on PCB electrodes, including electroless, electrodeposition, and sputtering. The PCB manufacturing services offer different surface finishes as part of the standard fabrication process. The most popular type is the ENIG coating [10]. However, the primary purpose of these coatings is to improve the solderability and shelf life of PCB boards and can leave exposed copper at the edges of the pads [11]. On the other hand, electrodeposition of hard Au or Pt can result in fully coated electrodes with a higher surface roughness, which can increase the effective surface area of the electrode [12].

**Figure 3:**
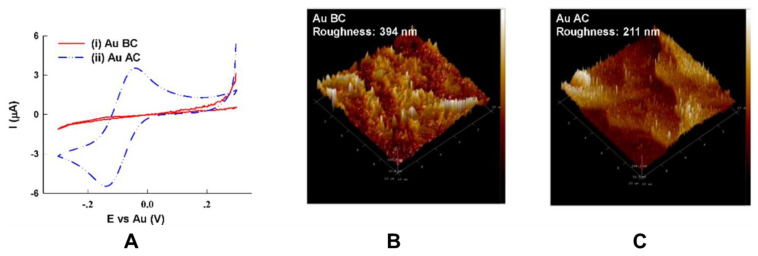
Comparison between Au-plated PCB electrode before and after cleaning: (**A**) cyclic voltammograms obtained from Au-Plated PCB electrodes in a PBS solution containing 4 mM K_3_Fe(CN)_6_ before cleaning (i) and after cleaning (ii); (**B**) atomic force microscopy of Au-plated PCB electrodes obtained before cleaning, and (**C**) after cleaning. The size of the atomic force microscopy (AFM) micrographs was not specified in the original figure. Reproduced from [13], Creative Commons Attribution License (http://creativecommons.org/licenses/by/4.0/).

Specifically, for electrochemical sensors, the surface physical characteristics and chemical properties of sensing electrodes are of great importance in reliable and accurate detection of a target analyte. Additional Au electroplating can result in a pore-free surface and improved electron transfer at the surface of the electrode. However, based on an observation by Dutta et al. [13], after electroplating gold on the surface of PCB electrodes, exposed Cu and an organic layer with a high content of C and O may remain on the surface, which makes the electrode electrochemically inactive. They have suggested a cleaning process using acetone, ethanol, and water followed by ultrasonication in an aqueous solution containing ammonium hydroxide and hydrogen peroxide t make the electrodes electrochemically active and decrease the surface roughness originated from the organic layer (Figure 3).

**Figure 4:**
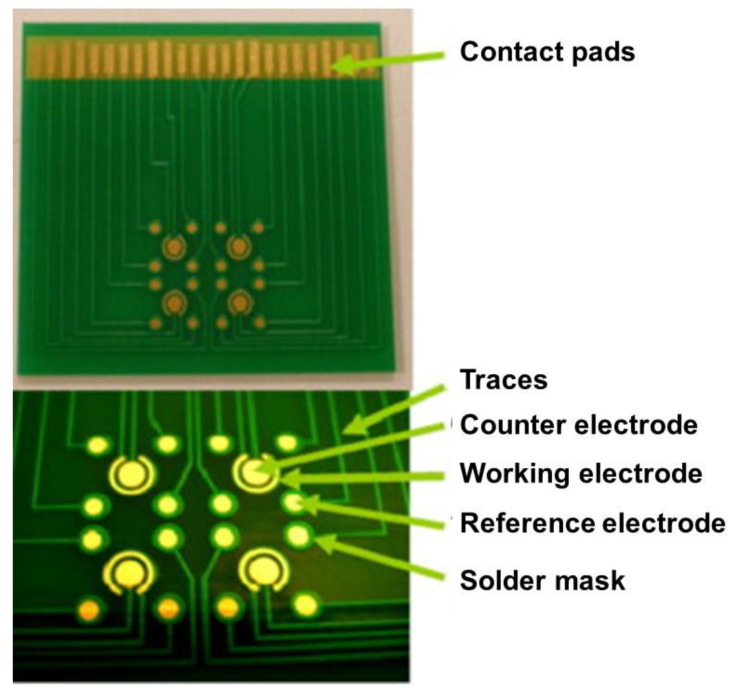
Photograph of the array chip fabricated using screen-printed Au ink on a PCB substrate. The diameter of the working and counter electrodes was 1 and 2 mm, respectively. The diameter of the outer and inner ring of the reference electrode was 4 and 3 mm. Reproduced with permission from [18], Copyright 2013 Elsevier.

Based on a study done by Evans et al. [14] on an Au-plated PCB electrode, the presence of chloride ions in the buffer solution could lead to the formation of a secondary electrochemical cell at the Au and copper interface. Comparing the electrodes’ surface during the amperometry analysis using a buffer containing chloride ions (PBS) and without chloride compounds (HEPES) showed that the inclusion of chloride ions results in the reduction and corrosion of gold. A layer of electroplated nickel between gold and PCB copper pads can improve the adhesion of the gold [15]. Furthermore, the nickel layer acts as a diffusion barrier to reduce the penetration of copper through gold and avoids the copper reaching the surface and becoming oxidized [16]. Besides, a solder mask can be extended to cover electrode edges to avoid the exposure of the copper at the edges [12,17]. Using an interesting approach, a low temperature curing Au ink was screen printed to form an array of sensing electrodes (Figure 4) [18]. One of the important parameters in developing reliable sensing electrodes is the thickness of the plated gold, which has not been considered in many reported PCB-based biosensors. A thicker gold layer has been found to generate a more stable characteristic cyclic voltammogram [19].

In electrochemical biosensors, the reference electrode maintains its potential with minimum current passing through it. Silver chloride (Ag/AgCl) electrodes are one of the widely used reference electrodes in electrochemical analyses. A similar reference electrode can be integrated on PCB-based electrochemical biosensors by electroplating or electroless deposition, an additional Ag layer, followed by chlorination using HCl solution [18,20,21], sodium chloride solution [22], or sodium hypochlorite [23]. In some sensing applications in which the true reference potential is not necessary, a Pt or Au coated PCB pad can be used as a pseudo-reference electrode [24]. In addition, PCB electroless immersion silver plating is a standard industrial process offered by manufacturers, which can be adopted to be chlorinated and used as a reference electrode in biosensors [25]. To reduce the sensor size, a single electrode can act both as reference and counter; however, this causes higher noise levels in the measurements [22].

## 4. Application of PCB-Based Electrochemical Sensors and Sensing Platforms

Various biosensors have been reported using PCB technology, which are summarized in Table 1. Glucose detection plays a key role in the diagnosis and management of diabetes mellitus. As a result, numerous enzymatic and non-enzymatic electrochemical sensors have been reported based on various fabrication methods, including screen printing [26,27,28], inkjet printing [29,30,31], and standard microfabrication processes [32,33,34]. Considering the availability and low manufacturing price of printed circuit boards, several PCB-based glucose biosensors have been reported recently. Glucose measurement is conventionally done using amperometry or cyclic voltammetry techniques through a three-electrode electrochemical cell.

Typically, the determination of glucose in a sample is done based on the glucose enzymatic reaction happening at the surface of the working electrode. The immobilization of glucose oxidase (GOx) enzyme on the surface of the sensing electrode affects the efficiency and sensitivity of the sensor. Although drop-casting the GOx on the Au-plated PCB electrode to develop glucose biosensor has been reported previously [15], more complex immobilization processes can improve the sensitivity and reproducibility of sensors. Dutta et al. [13] have formed a self-assembled monolayer (SAM) with activated carboxylic acid groups to covalently immobilize glucose oxidase on the surface of an Au-plated precleaned PCB electrode. A polymer matrix can also be used to immobilize the GOx on the sensing electrode. Kassanos et al. [20] used an additional layer of electropolymerized phenol red before drop-casting the GOx to develop an array of glucose-sensing PCB electrodes. After the immobilization process, the sensing electrode’s surface was coated with a polyurethane film to improve the dynamic range of the sensor.

To improve the sensitivity and selectivity of the glucose biosensors, the sensing electrode surface can be modified by various nanomaterials [35]. Carbon-based nanomaterials have been widely adopted in electrochemical sensing application due to wide potential window, low background current, and improved electron transfer rate [36]. The dependency of carbon nanotubes’ (CNTs) conductivity to surface absorbate and its ability to promote electron transfer have increased the use of these unique nanomaterials in developing a wide range of electrochemical biosensors [37]. Alhans et al. [38] drop-cast multi-walled and single-walled CNT dispersion solution on a PCB pad, which increased the electrochemical reactivity of the sensing electrode. The electrochemical impedance spectroscopy (EIS) results showed a decrease in electrodes resistance values and, consequently, a higher electron-transfer rate after the deposition of carbon nanotubes. The CNT working electrodes were modified by drop-casting GOx to form a low-cost PCB-based glucose biosensor.

Similarly, Li et al. [18] used a dispersion solution of CNT, polyvinylimidazole-Os (PVI-Os), enzyme (glucose or lactate oxidase), and chitosan composite sensing material to detect glucose and lactate electrochemically. Chitosan is a widely used biocompatible polymer in enzyme immobilization due to its high permeability toward the water and good adhesion [39]. As an electron mediator, PVI-Os improve the electron transfer while minimizing the enzyme leakage. The mentioned composite was dropped on an array of SAM-modified screen-printed Au electrodes to form glucose and lactate biosensors.

**Figure 5:**
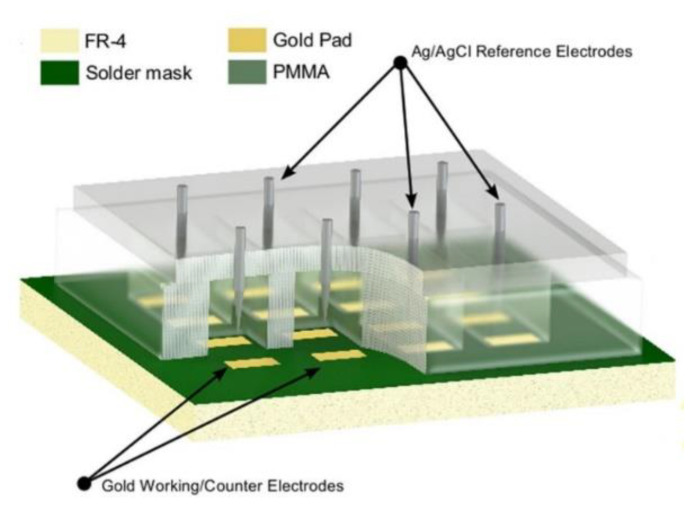
A three-dimensional representation of the fabricated prototype PCB-based sensor for the detection of tuberculosis using an enzyme-linked immunosorbent assay (ELISA). Reproduced from [41], Creative Commons Attribution License (http://creativecommons.org/licenses/by/4.0/).

An important area that low-cost biological sensors have attracted attention is point-of-care (PoC) diagnostics of disease caused by various bacteria [50]. Tuberculosis (TB) is an infectious disease caused by Mycobacterium tuberculosis bacteria, which is considered a concerning global health-related issue [51]. Commercially fabricated PCB sensors can be employed to fabricate a low-cost biodetection system for PoC diagnosis of tuberculosis. Evans et al. [41] reported a PCB-based amperometric electrochemical sensor for the detection of tuberculosis using enzyme-linked immunosorbent assay (ELISA), which outperforms the standard colorimetric ELISA technique in terms of limit of detection. The working and counter electrodes were fabricated on the PCB, the reaction well was formed on top of the PCB using polymethyl methacrylate (PMMA), and an external Ag/AgCl reference electrode was introduced to the system (Figure 5). The capture antibodies were covalently localized on the Au-coated PCB sensing electrodes using thiol linkage. The detection of interferon-gamma (IFNγ) as a biomarker using the proposed ELISA system was done by electrochemical detection [41,52].

According to the World Health Organization, foodborne illnesses caused by bacteria, such as *Salmonella typhimurium, Escherichia coli, Campylobacter,* and *Vibrio cholerae*, are a critical issue for public health [52]. Therefore, rapid and reliable detection of such pathogens plays a crucial role in the discovery of contaminations and controlling disease outbreaks. Nandakumar et al. [22] have developed a low cost PCB-based impedimetric sensor to detect *Salmonella typhimurium*. The sensing electrode was modified with *S. typhimurium*-specific antibodies. The infected samples can be distinguished by an increase in the impedance value resulted from the binding of the pathogens to the surface of the electrodes. A similar sensor structure was adopted by Dutta et al. [42] to detect *Streptococcus mutans* using a commercially fabricated PCB board.

Using a different approach, a PCB-based impedimetric sensor for the detection of *Salmonella* was reported by Wang et al. [43], in which the bacteria was selectively conjugated with magnetic and gold nanoparticles to form enzymatic bacteria. Next, the bacteria were employed to catalyze urea, which results in a decrease in the impedance of the sample.

One of the earliest applications of a PCB-based biological sensor was in molecular diagnostics, which was reported by the researchers at Motorola Inc. [46,53]. To electrochemically detect nucleic acids, the surface of the Au-plated PCB microarray was coated with DNA capture probes using a self-assembled monolayer (SAM). After the unlabeled nucleic acid targets were immobilized on this layer, ferrocene-modified nucleotides were introduced to the system as a signaling probe to form a sandwich complex. The SAM layer avoids non-specific binding of the electroactive species to the surface of the microarray while making the oxidation ferrocene-labeled adenosine derivative possible through the electron exchange with the gold. Later, this work led to a commercial sensor called eSensor^®®^ produced by Motorola Inc. for nucleic acid target detection and genotyping. This sensor is composed of an array of gold working electrodes, a gold counter electrode, and an Ag/AgCl electrode, which can be accessed through connectors at the edge of the board. PCB-based DNA detection platforms with integrated microfluidic systems have been reported using this commercial PCB-based biological sensor [54,55].

Gassmann et al. [47] reported on a DNA detection chip with an integrated microfluidic system, which was capable of performing polymerase chain reaction (PCR). The PCR process was done by cycle heating using copper traces on the PCB based on the Joule heating concept. Two separate PCB boards, one with microchannels and the other with electrodes, were fabricated separately and stacked together to form the DNA chip. In addition to temperature cycling, Tseng et al. [21] incorporated a PCB-based sensor on their platform to detect methylene blue. During PCR amplification, the methylene blue concentration decreases due to the binding to double-stranded DNA (ds-DNA) and single-stranded DNA (ss-DNA). The concentration of the methylene blue can be monitored using cyclic voltammetry and the fabricated PCB-based electrochemical setup.

**Figure 6:**
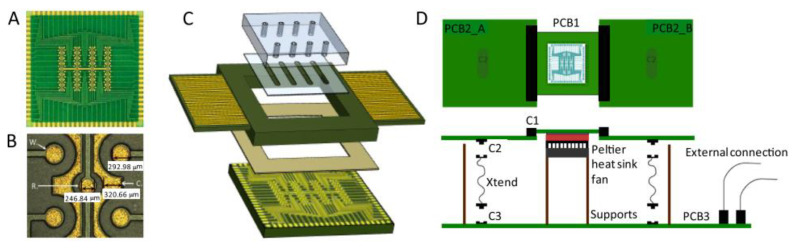
PCB based sensing platform developed for electrochemical detection of cancer-related mRNA markers: (**A**) Electrode arrayed developed using PCB technology; (**B**) magnified image of the PCB electrodes; (**C**) schematic the sensor along with the microfluidic system, and (**D**) fully assembled biosensing platform. The PCB-based chip was a square with a side length of 24.6 mm. The diameter of the working, reference, and counter electrodes was 300, 250, and 250 μm, respectively. Reproduced with permission from [48]. Copyright 2016 Elsevier.

Another interesting application of a PCB-based sensor in molecular diagnostics was showcased by Acero Sánchez et al. [48] for breast cancer-related mRNA markers. The proposed platform was composed of a PCB array with 64 Au-coated individually addressable electrodes in conjunction with an integrated PMMA-based microfluidic system (Figure 6). The sensing electrode surface was cleaned using acetone, isopropanol, and water, followed by oxygen plasma treatment. The presence of O_2_ removes the remaining organic materials, while the Au provides a fresh gold surface [56]. The integration of microfluid systems with PCB technology resulted in the emergence of the lab-on-PCB concept [57]. We suggest that interested readers refer to the review paper by Moschou et al. [58].

Cytokine, interleukin-12 (IL-12), is a biomarker found to have elevated ranges in patients diagnosed with an autoimmune disease called multiple sclerosis (MS) [59]. Bhavsar et al. [23] developed a robust PCB-based sensor to detect this protein biomarker by immobilizing anti-IL-12 antibody on the surface of an Au-plated PCB sensing electrode and performing electrochemical impedance spectroscopy. The proposed method reduces the detection time to 90 s with an ultra-low limit of detection (<100 fM).

**Figure 7:**
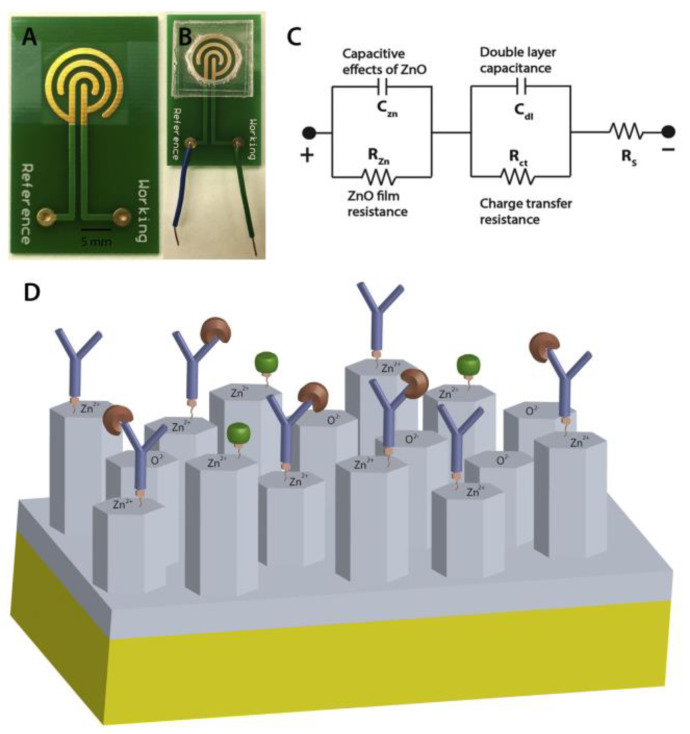
PCB based sensor developed for detection of Troponin-T as a cardiac biomarker: (**A**) PCB with electroplated gold electrodes and ZnO sputtered sensing site; (**B**) assembled sensor platform; polydimethylsiloxane (PDMS) manifold confines sample fluid on the ZnO sputtered sensing site; (**C**) electrical circuit model of the sensor, and (**D**) schematic of Troponin-T immunoassay on the nanocolumnar ZnO surface. The width of all the electrodes and the gap between each was 1 mm. Reproduced with permission from [49], Copyright 2016 Elsevier.

PCB sensing electrodes sputter-coated with zinc oxide (ZnO) has been reported to anchor capture antibodies [49]. Troponin-T is a cardiac biomarker that can be found in the bloodstream of patients with myocardial damage. The capture antibody was attached to the ZnO-modified PCB electrodes (Figure 7). The changes in the electrochemical impedance after capturing troponin-T by the capture antibody was used to detect the level of this protein.

The detection of Troponin-I, another cardiac regulatory protein, using a PCB-based sensing platform, has been reported by Lee et al. [60]. However, the sensing unit itself was fabricated using standard microfabrication processes and attached to the PCB later. Although the fully PCB-based sensors provide advantages of integration of electronic measurement systems without implementing additional connection strategies, this work is one of the great examples to see how a sensing platform fabricated with different methods can be easily integrated into a PCB board. While the connection of the miniaturized sensor to the outside world remains challenging [61], PCB platforms can offer a reliable alternative approach to overcome this problem. Several studies have benefited from the advantages of PCB technology to provide an interface for their biological sensor to be connected to electronics [9,62,63,64,65].

Recently, flexible PCB technology has gained attention in the fabrication of biosensing platforms. The wide viability of manufacturing services, low weight, and mechanical flexibility of this technology make it a promising candidate to develop wearable biosensing devices. However, the flexible PCB can be adopted to develop miniaturized thin wearable measurement systems to be used in conjunction with microfabricated biosensors [66,67]. In addition, flexible PCB itself can be used as the backbone of the sensor. For example, Pu et al. [40] developed a glucose sensor for the detection of hypoglycemia in interstitial fluid (ISF) on a polyimide substrate using flexible PCB technology. They employed inkjet printing as an interesting approach to modify the electrode surface with graphene. The advantages that such technologies offer, in combination with a well-established and rapidly growing PCB manufacturing industry, make this approach a great alternative fabrication method for the implementation of various biosensors.

## 5. Conclusions and Outlook

PCB technology offers an alternative, low-cost approach for the fabrication of various sensors. This approach facilitates the transition of prototyped sensors to the market and end-users due to the preexisting manufacturing industry that advances rapidly. In addition, the integrability of fluidics and electronics with PCB-based biosensing platforms makes them a great candidate for standalone point-of-care diagnosis systems.

Although PCB technology shares a lot of fabrication methodologies with microfabrication processes, it offers additional advantages in terms of long-lasting and thriving PCB manufacturing industries. This facilitates the adoption of PCB-based biosensors by the market and end-users. Furthermore, the similarity in the process opens up new opportunities to adopt already investigated biosensors’ designs and implement it on a PCB board to reduce the fabrication cost and promote commercialization possibilities. For example, novel materials, such as carbon nanotubes [68], reduced graphene oxide [69], metal nanomaterials [70], metal oxide nanoparticles [71], can be used to develop novel PCB-based biosensors with improved sensitivity and selectivity.

On the other hand, standard microfabrication processes outperform the PCB technology in terms of minimum feature size. However, the rapidly growing necessity for miniaturization of electronic systems pushes this industry toward improving this limitation. Besides, the use of copper, which is not usable in the electrochemical analysis, imposes additional modification steps for the development of reliable biosensors. To overcome this challenge, novel low-cost fabrication methods, such as inkjet printing [3] and screen printing [72], can be used along with PCB technology. In addition, further investigation into the electrochemical characterization of standard PCB pad finishes offered by the current industrial processes may lead to a promising substrate to perform electrochemical analysis.

Given the wide variety of target analytes and inherently different fabrication and detection methods utilized by the reviewed reports, the comparison between the sensors from a bioanalytical standpoint was beyond the scope of this review. However, interested readers can refer to multiple published review papers dedicated to electrochemical detection of a specific target analyte using specific detection methods and materials [50,73,74,75,76].

Overall, this review shows the capabilities of PCB technology as a reliable method to develop electrochemical sensors using different electroanalytical and bioanalytical approaches. The diminishing manufacturing price of PCBs due to the rapid growth of the electronic industry provides opportunities to adopt this technology for the fabrication of affordable disposable electrochemical sensors for point-of-care applications. Besides, the recent advancements in flexible PCB technology makes PCB-based sensors a promising candidate for detection in conditions that mechanical flexibility and total sensor weight is critical (e.g., wearable devices).

## Figures and Tables

**Table 1:** The summary of the reported printed circuit board (PCB)-based electrochemical biosensors.

PCB Pads Modification	Sensing Electrode Surface Modification	Target Analyte	Detection Method	Ref.
Electroplated Au	GOx ^1^	Glucose	Amperometry	[13]
Electroplated Ni, Au	GOx	Glucose	Cyclic voltammetry	[15]
Screen-printed Au	CNT ^2^, GOx/LOD	Glucose, Lactate	Amperometry	[18]
ENIG ^3^, Electroplated Au	Red phenol, GOx	Glucose	Amperometry	[20]
Au	CNT, GOx	Glucose	Amperometry and EIS ^4^	[38]
Electroplated Ni, Au	Graphene, Au NPs ^5^, GOx	Glucose	Amperometry	[40]
Electroplated Au	Antibody	*Mycobacterium* *tuberculosis*	Amperometry	[41]
Electroplated Ni, Au	Antibody	*Salmonella typhimurium*	EIS	[22]
Electroplated Au	Antibody	*Streptococcus mutans*	EIS	[42]
Electroplated Au	-	*Salmonella typhimurium*	EIS	[43]
Electroplated Ni, Au	Antibody	*Salmonella typhimurium*	EIS	[44]
Electroplated Au	Antibody	IFN-γ ^6^	Amperometry	[14]
Electroplated Au	Antibody	IFN-γ	Amperometry	[45]
Electroplated Ni, Au	Antibody	Interleukin-12	EIS	[23]
Electroplated Au	DNA probes	DNA	Sweep voltammetry	[46]
Electroplated Ni, Au	DNA probes	DNA	Square wave voltammetry	[47]
Electroplated Ni, Au	DNA probes	mRNA markers	Amperometry	[48]
Electroplated Au	ZnO, antibody	Troponin-T	EIS	[49]
Electroplated Ni, Au	-	Methylene blue	Cyclic voltammetry	[21]

^1^ Glucose oxidase, ^2^ Carbon Nanotubes, ^3^ Electroless nickel immersion gold, ^4^ Electrochemical impedance spectroscopy, ^5^ Nanoparticles, ^6^ Interferon-gamma.
